# Integration of research and practice to improve public health and healthcare delivery through a collaborative 'Health Integration Team' model - a qualitative investigation

**DOI:** 10.1186/s12913-016-1445-z

**Published:** 2016-06-22

**Authors:** Sabi Redwood, Emer Brangan, Verity Leach, Jeremy Horwood, Jenny L. Donovan

**Affiliations:** School of Social & Community Medicine, University of Bristol, Canynge Hall Bristol, 39 Whatley Road, Bristol, BS8 2PS UK; National Institute for Health Research, Collaborations for Leadership in Applied Health Research and Care West (NIHR CLAHRC West), 9th floor Whitefriars, Lewins Mead, Bristol, BS1 2NT UK

**Keywords:** Research and healthcare collaborations, Coproduction of healthcare knowledge and practice, Integrated knowledge translation

## Abstract

**Background:**

Economic considerations and the requirement to ensure the quality, safety and integration of research with health and social care provision have given rise to local developments of collaborative organisational forms and strategies to span the translational gaps. One such model – the Health Integration Team (HIT) model in Bristol in the United Kingdom (UK) - brings together National Health Service (NHS) organisations, universities, local authorities, patients and the public to facilitate the systematic application of evidence to promote integration across healthcare pathways. This study aimed to (1) provide empirical evidence documenting the evolution of the model; (2) to identify the social and organisational processes and theory of change underlying healthcare knowledge and practice; and (3) elucidate the key aspects of the HIT model for future development and translation to other localities.

**Methods:**

Contemporaneous documents were analysed, using procedures associated with Framework Analysis to produce summarised data for descriptive accounts. In-depth interviews were undertaken with key informants and analysed thematically. Comparative methods were applied to further analyse the two data sets.

**Results:**

One hundred forty documents were analysed and 10 interviews conducted with individuals in leadership positions in the universities, NHS commissioning and provider organisations involved in the design and implementation of the HIT model. Data coalesced around four overarching themes: ‘Whole system’ engagement, requiring the active recruitment of all those who have a stake in the area of practice being considered, and ‘collaboration’ to enable coproduction were identified as ‘process’ themes. System-level integration and innovation were identified as potential ‘outcomes’ with far-reaching impacts on population health and service delivery.

**Conclusion:**

The HIT model emerged as a particular response to the perceived need for integration of research and practice to improve public health and healthcare delivery at a time of considerable organisational turmoil and financial constraints. The concept gained momentum and will likely be of interest to those involved in setting up similar arrangements, and researchers in the social and implementation sciences with an interest in their evaluation.

## Introduction

The imperative to close the translational gaps between scientific research evidence and routine practice in the delivery of healthcare is particularly pressing in financially straitened times.

In advanced healthcare economies, the drive to exploit the potential of scientific innovation to improve quality through new approaches to prevention, diagnosis and treatment of diseases, and decrease costs by integration between services has given rise to a range of new organisational partnerships [1–3]. Such collaborative partnerships bring together the producers (e.g. academics and researchers) and users (e.g. service leads, policy makers, healthcare professionals) of research findings. In the United Kingdom (UK), the systematic integration of research into clinical practice and organisational routines is promoted so knowledge can be coproduced [4]. This approach is based on the accumulating evidence that research translation and implementation are contextually situated and complex, rely on multiple professionals and organisations, and involve multifaceted, iterative, and often unpredictable processes [5–7]. Coproduction of knowledge can be facilitated through meso-level (organisational) partnerships across professional and organisational boundaries, using new organisational forms [8]. While there is an increasing number of descriptions of these partnerships and their variants, a recent international scoping review [8] has highlighted a major gap in knowledge about the social and organisational processes underpinning their workings, and a lack of empirically grounded theoretical development.

The aim of this article is to describe the development and establishment of micro-level ‘operating units’, or Health Integration Teams (HITs), of a locally evolved structural partnership of National Health Service (NHS) organisations, local authorities, patients and the public, and universities, to foster collaboration across its stakeholders and generate health improvements for local populations through integrated working. Based on data from contemporaneous documents and reflections of participating key informants, we document the development of the HIT model and set it within the context of the coproduction of healthcare knowledge in the English health and care economy, and of the emerging organisational models to facilitate it.

## Background

The HIT model was developed following the formation of a collaborative partnership, the ‘Bristol Health Partners’ (BHP) in the West of England in 2011–12. This partnership, BHP, was a locally driven initiative by leaders of NHS organisations, a city council (local government with responsibility for a range of public services) and two universities who decided to build a partnership following an unsuccessful attempt to win competitive funding to establish a Collaboration for Leadership in Applied Health Research and Care (CLAHRC). CLAHRCs were established in 2008 in several regions in England as networks of research partnerships for applied health research and the translation of research findings into improved patient outcomes, supported by central government funding. The failure to attract funding led to a re-evaluation of the current arrangements for health research and knowledge translation, resulting in the BHP partnership and the subsequent development of the HIT model. The original plan was to emulate the Academic Health Sciences Centres (AHSCs) which were well established in North America and Europe including London, Cambridge and Manchester [9]. These centres combine the delivery of services to patients with high levels of research and teaching through sophisticated collaborative mechanisms between universities, hospitals and, in some cases, primary care based organisations. Their focus was on biomedical research in specific clinical disciplines, hospital-based services and early stage translation. However, the local context with research expertise particularly in population health linked to commissioning and public health, was a poor fit with the AHSC model. This article describes how the dominant biomedical discourse about knowledge translation was revised to formulate a new strategy with a focus on patient-centred integration across health and care pathways. The HITs which were set up from 2012 onwards and numbered 19 by October 2015, clearly reflect this re-visioning of knowledge translation, operating in areas related to public health, long-term conditions and cross-sectorial working (see Table 1). For a detailed description of the individual HITs see Bristol Health Partners, 2016 [10].

**Table 1:** Health Integration Teams, their aims and accreditation

Month of accreditation	HIT title	HIT aims
*July 2012*	Bristol bones and joints	• Harness evidence-based practice and associated research to fill knowledge gaps • Improve care pathways and outcomes for patients with musculoskeletal conditions
	Avoiding hospital admissions	• Reduce complexity in the local urgent care system • Optimise the productivity and efficiency of existing and new interventions
*December 2012*	Sexual health improvement	• Transform services to improve sexual health for the people of Bristol and the South West
	Improving care in self-harm	• Examine the care pathway, and utilise knowledge, expertise and resources to achieve the highest quality evidence based patient care and treatment for people who harm themselves
	Dementia	• Deliver dementia-friendly communities and services based on the highest quality evidence • Conduct world-class research to achieve the best quality of life for people and families living with dementia
	Supporting healthier and inclusive neighbourhood environments	• Use science, community voices and innovation to establish Bristol as a healthy city • Reduce health inequities • Closely align city development with health, well-being, social inclusion and green city aspirations
	Respiratory infections	• Improve the management of patients at every stage of their illness and care • Use NHS resources as efficiently as possible
	Retinal outreach, integration and research	• Implement research-driven service delivery • Engage the patient voice, staff and commissioners in developing those services
*July 2013*	Child injury	• Help Bristol set the national standard for integrating prevention, care and rehabilitation across children's trauma services
	Parkinson's and other movement disorders	• Develop whole system partnership working for movement disorders across the Bristol, North Somerset and South Gloucestershire region • Develop a high quality, high impact, internationally-recognised system for Parkinson's and other movement disorders
*December 2013*	Chronic Kidney Disease	• Improve outcomes for patients with kidney disease in the Bristol area through: prevention, patient care, education & research
	Bristol network for equality in early years health and wellbeing	• Focus on antenatal care to children aged seven • Achieve improvements in oral health, nutrition, and social and emotional wellbeing
*July 2014*	Active people: promoting healthy life expectancy	• Encourage the adoption of physical activity and other health behaviours among older age groups in order to improve their overall health during their later years
	Addictions	• Maximise the use of the resources already available to reduce substance-related harm
	Integrated pain management	• Improvements in performance, productivity and efficiency by ensuring that our research programmes and expertise in the management of chronic pain are integrated into care
	Bristol immunisation group	• Develop an outstanding immunisation service • Lead research on immunisation development and provision, responsive to vaccine preventable infectious disease outbreaks • Use innovative technology to enable people to be better informed regarding their own (and their children's) vaccinations
	Psychological therapies in primary care	• Improve uptake of, access to, and outcomes for effective psychological therapies
	Improving perinatal mental health	• Improve the identification and subsequent care of parents with poor mental health before and following the birth of their child
*July 2015* (Expressions of interest)	Cancer, Chronic eye conditions, Psychosis, Eating disorders	

Politically, the period of time during which these developments took place was characterised by acute uncertainty following a new coalition government’s proposals for a top-down, highly complex NHS re-organisation which passed into law in March 2012. This coincided with the establishment of BHP in April 2012 and the issuing of the first invitations to become accredited HITs. The re-organisation presented those trying to build new partnerships with particular challenges since established NHS bodies such as the Strategic Health Authorities who provided leadership at system level were abolished, and new bodies such as the Clinical Commissioning Groups (CCGs) were established as membership organisations led by primary care doctors or General Practitioners (GPs) [11]. The term ‘commissioning’ is specific to England and refers the strategic planning and purchasing of health care services for the local population. In England, where there is significant centralised direction and performance management from the Department of Health [12] commissioning also involves the implementation of national policy within the context of local needs and resources. Another change resulting from the reforms was the transfer of public health functions from the NHS to local government and the formation of a new body, the Health and Wellbeing Boards to link GP commissioners to local government and to provide a forum better to link commissioning plans for health and social care services.

The implications of the reforms, as Ham et al. [11] point out, was a leadership vacuum at the system level as the leadership previously provided by the Department of Health centrally and the Strategic Health Authorities regionally was distributed between several organisations, each overseeing a part of the NHS without having system level oversight or responsibility. Similarly, at local level where system leadership between commissioners, providers, local authorities and other partners was vital at a time when new integrated models of care were required to meet growing demands on health and social services, it was missing, in part due to current senior leaders having responsibility for individual organisations, rather than the system. The reforms also resulted in the fragmentation of commissioning and a loss of population based commissioning because the responsibility for procuring services was split between CCGs, NHS England and local government. The drive to develop and implement the HIT model was a response to these consequences and to mitigate the effects of lack of system leadership and the risk of fragmentation. It was also an initiative to promote evidence-based practice in commissioning and service delivery and a forum for integration.

Drawing on the principles of theory-based evaluation [13–15], this study sought to provide empirical evidence about how the HIT model emerged; identify the theory of change and social and organisational processes underlying the coproduction of healthcare knowledge and practice; and elucidate the key aspects of the HIT model for future development and translation to other localities.

## Methods

Using a qualitative design, the research team accessed, collated and analysed contemporaneous documents, and conducted interviews with individuals who were leading the development of the HIT model. Methods and findings are described below, adhering to criteria for reporting qualitative research [16].

### Documentary analysis

The research team was given permission to access documents belonging to BHP, the organisation accrediting HITs, produced between September 2010 and August 2014, describing the development of the HIT model and setting up of individual HITs. All documents were anonymised before being imported into a qualitative data management software package (QRS-NVivo 10) for coding and analysis. Procedures associated with Framework Analysis [17, 18] were used to produce summarised data for descriptive accounts. These accounts traced the processes though which the partner organisations collaborated and established the structures and facilitating conditions for the model, and were used to complement the data generated through interviews with key informants.

Documentary materials related to the establishment of the HIT model and individual HITs, and were collected from the point at which expressions of interest were submitted, the application process and later set-up period, and so varied depending on how long the HIT had been in existence. They included discussion papers, application forms, feedback documents, progress reports and meeting notes. An initial coding-frame was drawn up to reflect this. Documents tracking the strategy were organised chronologically and coded inductively.

### Interviews with individuals who were leading the development of the HIT model

Interview data were collected face-to-face or via telephone from individuals in strategic leadership roles who formed the BHP steering group and were involved in the design and implementation of the HIT model. Participants volunteered to take part following an explanation of the rationale for the study at a meeting and subsequent distribution of written study materials. Interviews were digitally recorded and transcribed in full. Interview questions were open and broad, seeking information on participants’ roles in the development of the HIT concept and their recollections, followed by each participant’s perspective and understanding of how the model was supposed to work and produce beneficial outcomes. Fieldwork notes, debriefing notes and analytical memos were kept to assist in the analysis which was based on the principles of the constant comparative method [19]. This involved initial coding, the forming and refining of categories, searching for negative evidence and comparison across each stage of the analytic development of explanatory concepts which were complemented by the analysis of the documentary data. Data management, coding and categorisation were supported by QRS NVivo software.

## Results

In total, 140 documents were included in the analysis, with 10 interviews with individuals in senior leadership positions at two universities, one NHS commissioning organisation, and four NHS provider organisations. They included academics and clinical-academics who went on to become HIT directors, and senior managers. VL, EB and SR collated, coded and analysed the documentary materials; SR collected and analysed the interview data. Despite the heterogeneity of the data, four overarching concepts emerged. These permitted the development of an initial implicit theory of change and clarified the underlying logic of improvement while making explicit some of the mechanisms that were considered crucial in producing desired outcomes. These concepts were (1) ‘whole system’ engagement, (2) collaboration, (3) integration and (4) innovation. First we will describe the genesis of the model and its context, and then move on to elucidate each concept.

### HIT model genesis

The model’s antecedents go back to 2010 when the BHP partnership was formed across NHS organisations, city council and universities to promote research and innovation in the area because of set-backs in attracting research funds and the perception that local organisations were less well connected than in other areas. As one participant said, ‘we decided that we would get our act together in terms of research and other relationships’ (Participant 1, academic). Some progress was made but ‘[the steering group] really wasn’t moving the agenda forward’ (Participant 2, clinical academic). Consequently, an independent consultant was commissioned to explore possible options for a model closely aligned to the group’s ambitions and to provide an in-depth analysis of the potential strengths, opportunities, political and economic risks, and organisational and governance models.

The model that had gained currency at the time was the Academic Health Sciences Centre (AHSC) as outlined previously, which was focused on hospitals and medical schools, biomedical research and clinical disciplines. The participating organisations were closely connected through partnership agreements, common standards and goals, and an overarching identity. The UK Department of Health had accredited five English AHSCs in 2009 to integrate research, teaching and service strategically and operationally to deliver ‘a whole which is greater than the sum of the parts’, and to form a recognised elite attracting investment research and innovation. This was the direction of travel the consultant was recommending for BHP:

*‘He was very much of the mind-set of an AHSC where you had pre-clinical biomedical sciences with early stage translation and that was the model he was used to. He based some of his early documents around the King’s model and even called them Clinical Academic Groups.’* (Participant 2, clinical academic)

The Clinical Academic Groups [20] were ‘the key building blocks of all English AHSCs [and] are specialty-level, cross-organisational groups’ (Development period, 2011), and clearly focused on biomedical research in specific clinical disciplines, hospital-based services and early stage translation. These groups were seen as the leaders for research, translation and innovation:

*‘[The consultant] was quite insistent that the most important things were strong leadership, and so clinical academics were seen to be strong leaders, and that it should be around translation. Everybody always talked about translation as if it was just Type 1 [early stage basic research] translation. There was no real consideration for Type 2 [development of new approaches and technologies] really.’* (Participant 1, academic)

The AHSC model initially had strong support because of the emphasis on partnership working. However, there was a growing awareness that there was a lack of alignment with the more broad-based aspirations for wider collaboration and integration across the health and care landscape in the local area. The following two excerpts illustrate this mismatch:

*‘It felt too clinical. It felt too hospital orientated. It didn’t seem to draw on primary care, the prevention agenda, public health, the kind of things that we research in Bristol, but it also didn’t represent the ethos around what we were trying to do.’* (Participant 6, academic)

*‘[The report] does not currently fully represent or engage with Bristol’s key strengths in population health sciences research, clinical translation, social and community services and public health, or the City Council’s priorities. These issues provide an impetus for a new vision to drive partnership across the city.*’ (Document from development period, 2011)

An alternative model was developed from within BHP that addressed ‘key public health issues through the integration of research and practice’ (Development period, 2011). Its aims were to build on existing collaborations and develop new partnerships between commissioners, primary care and public health professionals, allied health professionals, specialist clinicians, basic scientists and health service researchers with active involvement of patients and the public. This model was more attractive locally because it suggested ways of working across the whole health and care economy, involving a much wider range of stakeholders, rather than privileging particular clinical disciplines. Furthermore, it found resonance among a wide range of local academics and researchers who had developed programmes of population-based research which was more closely aligned to the new model than the clinical, often laboratory based research carried out in AHSCs. As the alternative model gained currency, ideas about how these aspirations could be delivered began to emerge and the first references appear to what became the models’ ‘operating units’, the HITs. Their purpose was defined as:

*‘[integrating] research and clinical delivery in agreed ways between the (…) partners, for example by establishing new or more efficient care pathways, introducing or evaluating commissioning around the primary/secondary interface, and more effective, efficient and acceptable ways of treating patients. There would need to be detailed negotiations between Trusts [any NHS organisation such as hospitals or primary care organisations] and Universities to ensure integration.’*

The BHP steering group then designed a process of accreditation, encouraging key individuals and their organisations to identify common goals and formulate joint action. Thus the group created a common structure for collaborative working across organisations and sectors, and - through the accreditation methods - reinforced the behaviours and processes they wished to encourage. There were no prescribed themes or areas of practice around which HITs were expected to form. Instead each HIT was able to develop its own collective identity, mobilise commitment and align with HIT members’ values and aspirations.

### ‘Whole system’ engagement

The first of the overarching concepts emerging from the data refers to the process of actively seeking to engage organisations and people who play a part in the health and care economy related to the long-term conditions or public health issue at the centre of the HITs being formed. Those engaged included provider organisations, commissioning organisations, professionals/staff in these organisations, public(s) and service users and carers, other public sector organisations (for example, local councils and health and wellbeing boards), and other non-NHS/social care organisations (for example, third sector organisations, industry). Achieving this kind of whole system engagement is challenging because individual organisations are accountable for their own performance rather than for their performance in relation to the system. In other words, each organisation is focused on providing the service or function it has been commissioned to provide with little incentive to consider the entirety of the provision as experienced by patients or service users. The proliferation of providers and the subsequent fragmentation of patients’ journey through the health and care system has been exacerbated by the NHS reforms in 2012, particularly because leadership and commissioning arrangements have been further broken up and senior officers continue to be accountable for the performance of their own organisation rather than that of the whole system.

As HITs were charged with the task of integration across a number of different boundaries within the whole health and care system, their membership proved to be a decisive factor in achieving some leverage on the very challenging problems being tackled. Applicants were required to explain who needed to be involved in the HIT and in what specific ways to achieve specific outcomes or goals.

This process-related theme of ‘whole system’ engagement provided a range of examples which highlighted the importance of creating a legitimate space where all those who had a stake in the public health or service delivery issue being addressed could voice their perspectives and the accountability demands that are often associated with these perspectives. Despite a longstanding commitment to engagement, involvement and networking across sectorial, professional and organisation boundaries to tackle shared problems, it would appear that facilitating such dialogue and interaction through structural partnerships made it easier for people to establish personal contacts and coalitions, and promoted joint action. Table 2 summarises the main features of this theme and provides illustrative data extracts.

**Table 2:** ‘Whole system’ engagement

Concept type:	Process related
Short definition:	Identifying and actively seeking to engage organisations and people who play a part in the health and social care economy related to the long-term conditions or public health issue at the centre of the HIT being formed.
Function	Data extracts
Dealing with challenging issues which cross organisational and sector boundaries.	*‘[Those coming together in the nascent HITs] realised they were grappling with different ends of the same issue and actually they had a lot they could share*’ (Participant 1, academic) *I think there’s great strength in having the whole system involved because that is how people’s lives work and that’s how we’ll get out of the mess we’re in.’* (Participant 7, senior commissioner)
Making research more relevant and deliverable.	*‘I bought into the notion that if you have service providers and commissioners and researchers all together, and then you design research which is going to meet the needs of the commissioners and the service, you’re more able then to deliver the findings of the research … So I just bought the notion that it would be more relevant and more deliverable. That’s why I supported it.’* (Participant 7, senior commissioner)
Key aspects	
Facilitated by the structured, iterative application process.	*‘We were delighted to hear that the work involved in putting the application together has already facilitated new connections and new conversations: this is exactly what we want to achieve.’* (Feedback document to HIT, 2014)
Creating a new space in which ideas could be discussed and explored without concerns about invading other organisations’ territory or individuals’ agendas.	*‘I think they have created conversations across the city and legitimised conversations between different organisations, not just academics and the service, but also between different parts of the service’.* (Participant 6, academic) ‘*Would we have succeeded without the HIT? The answer is I don’t know in all honesty. I would have thought we would have got to a certain level without question because of the energy and the drive that was being created. What the HIT’s done is mandated what we were doing and it has opened doors as a results of just having, not just of having a label, but of having an opportunity that has been mandated by the wider [health community]. That was really important and really was a step change in our speed of development.’* (Participant 5, HIT director, clinical academic)
Changing norms about who should be included.	*‘If I think of the 360 degree segments, I was probably covering around 90 degrees; maybe 100 degrees if we’re being optimistic, of the key people that we really needed to include in something that was going to look at such a challenging area. … Now I think I wouldn’t even conceive of doing something like this without including all the players, and we’re constantly thinking of other people we should include.’* (Participant 10, HIT director, clinical academic) *‘Our ambition is that this policy should underpin a culture shift such that PPI [Patient and Public Involvement] is embedded at all levels, including commissioning, decision making and policy rather than being limited to the logistics of service delivery and questions of patient satisfaction’.* (HIT application document, 2012)
Involvement of commissioners – going beyond previous collaborations between academics and large providers.	*‘What I think is really strong about the HIT model is that involves commissioners and when you go to other places and you look at what they’re doing, they tend to be less strong about having the whole system in the room. So typically you tend to see a lot of evidence of working with acute, but not necessarily mental health, not local authority and not commissioners.…. So I think that’s an incredibly important point that if you’ve got commissioners at the heart of it and in some of these leadership roles, it looks very different.’* (Participant 7, senior commissioner)
Meaningful and timely involvement and engagement of patients and public(s).	*‘So how do we infiltrate the real decision making spaces within the organisation so public voices are actually being heard where decisions are really being made?’ (Participant 3, academic)*

### Collaboration

The second process theme of collaboration refers to the development and embedding of methods that enable partnership working and co-production across HIT specific structures and procedures.

Sponsorship at the most senior level in one of the organisations forming a HIT, usually the employing institution of the HIT director, was seen as vital not just as a sign of organisational commitment to the HIT by providing time and resources to support its work, but also to proactively seek out opportunities for collaboration and integration (see Table 3). This suggests that connecting HITs to local resources was seen to be an important role of senior leaders in the health and care economy to facilitate new relationships and partnerships. While these relationships could be pre-existing or initiated through the HIT application and setup process, their quality and fruitfulness were contingent on factors such as ‘a common ideology’ of seamless and integrated care pathways and services. In order to create and support such a common ideology to serve the requirements for collaborative working, an infrastructure for sustained interaction, communication and exchanging information and knowledge between and across HITs and their collaborating organisations and individuals had to be developed.

**Table 3:** Collaboration

Concept type:	Process related
Short definition:	Development and embedding of methods that enable partnership working and co-production, involving HIT specific structures and procedures
Function	Data extracts
Breaking down organisational, sectorial and professional boundaries and silos by enabling effective communication about common goals	*‘…relationships are the most important thing in order to move anything together in a partnership to me. So the aspect of one area that has been successful was that the relationship with the commissioners was strong in the sense of common ideology. And mutual respect of each other’s tensions to deliver this, so when you understand that you can actually find a common goal that would achieve. So you’re getting commissioners, the spread of medics and allied health professionals together in order to just simply put the patient [in the centre] as doing the right thing for the right reasons and respecting each other’s tensions in order to deliver that.’* (Participant 5, HIT director, clinical academic)
Key aspects	
Requirement for sponsorship at the most senior level in one of the organisations forming a HIT.	*‘The HITs are all supposed to have executive sponsors from the organisation and it’s making sure that’s that a live relationship because those executives I think can really help the HITs but, you know, they’ve got to keep them in their mind all the time. Whenever anything comes across their desk they have to think “Oh, yeah, this might help my HIT.”’*(Participant 7, senior commissioner)
Building infrastructure for sustained interaction, communication and exchange of information and knowledge between and across HITs and their collaborating organisations and individuals.	‘*The working groups (…) will each be led by two individuals, encompassing academic and service leadership. These will both report to and work with the two thematic groups’.* (HIT application document, 2013) *‘This HIT will be embedded managerially within the Division of [relevant clinical sector at acute trust]. The Head of Division and the Divisional Manager will sit on the steering group. The steering group will meet monthly and report to the trust-wide [relevant clinical conditions] steering group, which has representation from across the trust, the commissioners and [regional provider]. The agenda of the HIT and the [clinical conditions] steering group correlate strongly and support clinical service delivery.* (HIT application document, 2012)

Several HITs had identified skills gaps within their teams and had taken steps to address these. For example, HITs had enlisted input and advice from third sector or public sector organisations whose remit included supporting engagement with commissioners, industry, or patients and the public. A review group comprised of public members was created with the aim that every HIT would ‘*have genuine PPI (Patient and Public Involvement) within their structures*’ (Participant 3, academic). There were varying degrees of previous PPI experience by HIT applicants and additional support was put in place:

*‘So the thing that came out of that was that we recognise the need to try and offer more practical support to the HITs. And that led to the creation of the PPI facilitator post (…).’* (Participant 3, academic)

As early HITs began to form and new collaborations emerged, it was important for mechanisms to be established which would create and foster conditions for collaboration across HITs to facilitate shared learning and to build capacity to address complex problems:

‘*All HITs are expected to coordinate their activities with other relevant HIT teams and this is particularly important in areas where a number of new HITs are developing.*’ (Feedback to HIT, 2014)

Indeed, many HIT applicants made reference to plans for such coordination by specifying and delineating their particular sphere of influence while emphasising the role and added value of ‘collaboration of collaborations’. Some HIT applicants also described strategies to facilitate improvements in collaboration which required an internal focus on the part of all partner organisations in order to “*establish confidence and capability to share data between organisations and agencies where this is in the patient’s interest (e.g. to evaluate need and outcomes of service provision)*” (HIT application, 2013) at an early stage. In other words, for organisations to be able to collaborate within a HIT or across other HITs, information needed to flow across organisational boundaries, requiring HIT management group members from different organisations to facilitate progress in this sensitive area, being responsible for engaging key individuals from their own organisations in the process:

*‘Management group members will liaise with information governance leads in their own organisations to facilitate collaboration and ensure an effective data sharing agreement can be established.’* (HIT application, 2013)

One HIT described a “*key strategic advantage*” of their 13 member executive board as being that “*these members in turn link with additional specific networks*”. The HIT planned to formalise the processes by which this collaboration mechanism would be implemented, and use their executive team as knowledge channels to/from other organisations. Apart from these more traditional channels for collaboration, several HITs also gave details of other structural mechanisms which could serve to support collaborative work. These included requiring dual leadership for each working group by ‘*two individuals, encompassing academic and service leadership*’ (HIT application, 2013); designating individuals who had dual NHS/academic appointments as theme leaders; embedding HIT structures within the managerial and service delivery structures with which the HIT sought to engage; and tying specific work-stream/theme groups structurally to cross-cutting themes such as evidence synthesis, inequalities, and PPI.

In summary, the second process-related theme provided some evidence of the procedures and structural conditions that were seen to support collaboration. The orientation of the strategies being employed was outward-looking to involve a wide range of stakeholders, and to gain visibility and legitimacy, to facilitate growth and attract the required expertise to develop relevant projects or work streams. Internally orientated strategies to build and strengthen the team responsible for the delivery of the work and to consolidate internal ways of collaborating were less evident in the data.

### Integration

This theme refers to a range of activities that are expected to lead to joint working across a range of spheres: service delivery, biomedical and population based/community research, data linkage and data intelligence. While these activities are process-related, integration was also seen as an outcome in terms of the integration of services across pathways in a unified system of health and social care, supported by interdisciplinary research bringing robust and high quality evidence to the care pathway from prevention to treatment and chronic condition management to palliative/end-of-life care. Notwithstanding existing accountability relationships that militate against fully integrated governance arrangements, the excerpt below addresses the importance of system-thinking:

*So …moving on beyond the language of collaboration and actually starting to see how that will work in practice and moving towards a position where organisations are operating in a framework which will encourage them to make decisions on the basis of system benefit and not organisational benefit. That’s a journey we’re still on … and the HITs are important in that regard because they are a material demonstration of how that will work in practice.* (Participant 8, senior NHS manager)

There are a range of examples in the documentary data that illustrate the integrative potential of HITs at various levels, from plans to develop specific patient-centred ‘one-stop-shops’ and integrated assessment clinics, to streamlining strategic oversight in their area of health and social care. In one long-term condition area changes which had occurred since the HIT’s formation, driven in part by the political context, had led to fragmented accountability, and ‘*a significant amount of duplication and overlap across the different meetings*’. Stakeholders sought a more appropriate way of ensuring work in the relevant chronic condition area continued to be developed and overseen. This was ‘*an increasing priority for commissioners in the light of staffing reductions within the local authority and within CCGs*’. The HIT was central to a proposed new local structure for work in the area, with various existing structures being subsumed or transformed. The objective was to reduce duplication while continuing to meet statutory requirements and those of the various stakeholders. The central role for the HIT was expected to both ‘*streamline current meetings*’ and ‘*add value to the system*’ (Restructure proposal from CCG, 2014).

Many HITs sought to drive integration by working with commissioners – both by facilitating a combined approach with coordinated priorities in areas where commissioning priorities were split, and by developing commissioning strategies to support delivery of integrated care.

Integration of information and data was another area of focus for HITs, with several teams envisaging their work informing the Joint Strategic Needs Assessments, and some had already started bringing together data sets to inform service design and delivery. One HIT was working with a local CCG to develop a system of integrated information support and advice to GPs, to facilitate implementation of the integrated pathway they were developing. Another HIT director saw the integration of services as a vehicle for geographical equality of access:

*‘The community we cover is quite widely spread, we’re not reaching out to our whole community with the same excellence that we reach within this building [specialist hospital]. There was a massive disparity of care and [access to research]. So what (…) the HITs have enabled us to do is to create a culture … that will engage commissioners, will engage the public, will engage the vertical integration of primary and secondary and tertiary care.’* (Participant 5, HIT director, clinical academic)

Another type of integration was related to the flow of information and feedback across the system, bringing together knowledge, experience and views from different groups of stakeholders, including those of patients and the public:

*‘…support three-way communication between communities, professionals and public health interests, through capacity building, providing an evidence base and knowledge translation. This will enable commissioners to be better informed by communities about the impact of their actions and to better support local need.’* (HIT application, 2012)

A senior manager involved in the genesis of the HIT model saw their inclusivity and longer time horizons as key factors in their integrative potential:

*‘So this is a fantastically sort of diverse range of teams working at different levels and different scales on a whole range of different issues, but unique in the sense that unlike the vast majority of other health partnerships that have kind of either explicitly or unofficially adopted a sort of clinical academic group type model, this one is much more inclusive. And I think it’s also in the longer term so much more powerful as an engine of driving integration.* (Participant 8, senior NHS manager)

The theme of integration contains elements of process-related aspects, but is predominantly outcome orientated in as much as the motivating force behind the work related to this theme was generating system-level change, especially in service delivery, and linking patient journeys from prevention to the provision of secondary/tertiary care services.

### Innovation

The second outcome-related theme refers to the successful exploitation of new ideas and novel ways of delivering services or interventions through the introduction or application of new approaches, usually to improve quality and decrease costs. This may include biotechnology products and IT solutions; collaboration with industry; new roles and/or service delivery models; and new insights through data linkage and data intelligence. Such data linkage and insights, which would be facilitated by the engagement, collaboration and integration elements of the HIT model, could have the potential to generate step-changes in the system:

*‘The hope is that they will potentially open up new ways to think about the sorts of interventions that will have real impact. So spotting an unknown correlation might unlock a whole different sort of area of policy interventions that we just don’t focus on at the moment.’* (Participant 8, senior NHS manager)

Expansion of relationships with industrial partners via HITs was another element of the model envisaged as supporting innovative work. HIT documents gave an indication of some of the specific innovations in development with industry partners, including novel ‘near patient’ testing technologies where investigations are carried out at the time of the consultation with rapid availability of results [21] for use where a swift diagnosis has important implications for treatment decisions, the development of technologies to facilitate access to services, and redesigning existing products to enhance safety. Partnerships with industry were also being drawn on to access resources for innovative research which would not otherwise be available: funding, directly from industrial partners, or via applications to commercial/industrial orientated grants; industrial partners’ specialist technology and facilities for research and development; and pools of staff who could fill capacity or expertise gaps.

Engagement and collaboration with other types of organisation were also seen as potentially supporting innovation, for example, partnering with third sector organisations already known locally for their innovative practice, or making new academic connections with a view to developing innovative interdisciplinary research. Many HIT applications included plans to pilot new interventions and services. These involved developing/tailoring decision support tools for primary care to provide local context-specific guidance, and promote agreed local strategies and pathways. A CCG collaborating with one HIT aimed to give ‘*greater flexibility to Primary, Secondary and Tertiary Care to work interactively and innovatively to develop new ways of working*’ (HIT application, 2014) in the HIT’s area. A ‘hub and spoke’ model to provide access to specialised services, which was developed drawing on a local centre of excellence, was actively being considered by the relevant Clinical Reference Group for early adoption as one of the regional pilots. Another HIT had used data collected as part of an innovative surveillance scheme to identify and address service provision shortfalls.

Highlighting the importance of collaboration between providers, commissioners and researchers in generating novel models of care, the following participant alludes to the complexity of implementing innovative change and the need for context-specific and sensitive evaluation:

*‘We are doing a whole bunch of real life experiments here, driven by the need to innovate our way out of difficulties. Let the researchers loose on answering the questions of ‘is this model working’? It is more subtle than ‘what works and what doesn’t work’, but what are the layers of decision-making and service change and institutions that are involved? How do they piece together to make a final product as it were? And how is that final product being perceived? Is it value for money, is it good quality care?’* (Participant 4, CCG senior manager)

HITs also developed innovative plans for education and training – for professionals, but also for patients, carers and the public. These plans included new ways of delivering training for front-line staff to improve both quality and access; facilitating evidence-based continued professional development, and inter- and cross-professional learning; and developing and disseminating materials to a broad range of members of the public and non-health professionals involved in caring for others, to support decision making about when and where to seek health care. Plans for innovation also included patients and the public, enabling them actively to contribute to novel products and processes through new media, knowledge cafes and workshops designed for children and young people.

The second outcome related theme offers some insights into how conditions for innovation were being fostered to generate locally appropriate solutions to health or healthcare delivery problems.

## Discussion

The aim of this study was to provide empirical evidence about the development of a locally-evolved model in cross-sector, cross-organisational and inter-professional working, and to develop a theory of change for the social and organisational processes underpinning its functions. Stripped of its specific local context, the theory of change we identified was that developing the specific processes of *‘whole system’ engagement* (identifying the key organisations and individuals who need to be involved in meeting joint objectives - in the case of the HITs this was patient-centred integration across health and care pathways) and *collaboration* (developing the most productive ways of working together across professional, disciplinary, sectorial and organisational boundaries) created the right conditions to produce the desired outcomes of *integration* (where organisations and individuals came together to produce a good or service that was joined up and fit-for-purpose) and *innovation* (where something new was created or new connections were developed with something that already existed). We have provided some specific examples of how these processes and outcomes were anticipated to work in the local Bristol context.

The HIT model was developed by a partnership of NHS organisations – providers as well as commissioners, two universities, a local council and including patients and the public to foster the co-production of knowledge and facilitate collaboration to generate health improvements for the local population. It shares many of the characteristics of the ‘communities of practice’ model, originally developed by Lave and Wenger [22] to describe practice-based professional learning, and later adapted by Wenger and colleagues [23] to the wider organisational setting to help conceptualise the management and mobilisation of knowledge by diverse groups for particular goals. The HITs are a new organisational form of structural partnership, designed to achieve linkages between knowledge requirements and knowledge production and as such can be described as ‘boundary organisations’, [24, 25] designed to facilitate collaboration and information flow between research and practice communities. The aim is to ensure that research is relevant to the local health and care economy, and that the use of evidence-based practices and local findings is increased. The collaborative processes described here that to bring together knowledge producers, knowledge users and the wider health and care community including patients and service users are closely related to what has been described as ‘integrated knowledge translation, broadly described as collaborations between researchers and decision-makers' [26] which builds on all participants’ capacity to value each other’s very different perspectives, providing a lever for system-wide approaches to sustainable change [27].

Although these types of partnerships are not new, the inclusion of commissioning bodies, and local government - which since 2013 has a wider remit for tackling the social and economic determinants of health – is unlike other knowledge mobilising organisations in the healthcare economy such as AHSCs. These typically involve large teaching hospitals and their associated clinical services, leading research intensive universities and their medical schools, and tend to focus on discovery and early phase translational work. Their operational units are typically organised around clinical academics in areas such as cardiovascular services, diabetes care or psychiatry. Although the AHSC model had initially found favour with a number of key players in the local partnership, the biomedical and clinical services focus was replaced by a broader perspective of population health sciences which underpinned a strategy of integration of care pathways including community services, the primary/secondary interface, public health and hospital care. Furthermore, the organisationally and professionally broad partnership was set up purposefully to gain leverage to effect population health gains both at pace and scale.

### Limitations

The mechanisms by which this new structural partnership model were thought to generate desired outcomes had to be identified retrospectively, based on documentary evidence and the recollections of key individuals involved in the design of the model. Given the time lag of up to four years since the initial meetings took place, some of the recollections may have been inaccurate and overlaid with more recent experience and insights. However, the interviewees’ accounts broadly concurred in terms of timelines and the content of key documents. Documentary evidence in the form of meeting notes and minutes was available and used to compare the verbal accounts, and again there were no significant disagreements on how the HIT model developed. However, the documents were not produced for the specific purpose of tracing the development of the model, and the interviewees’ accounts were inevitably partial, reflecting their perspectives and commitments, hopes and aspirations for the model they were putting in place. While overly positive presentations may have been problematic for a formal evaluation of the model, it was possible in the analysis to identify the ‘active ingredients’ of the model in a balanced way. Furthermore, participants were candid and open about areas where opinions differed among steering group members when the HIT model was first developed and the focus shifted from clinical and biomedical research to population-based research with a public health and service commissioning orientation. The documents relating to the specific HITs themselves such as application forms were highly structured and formal, and the content was presented in ways to appeal to the accrediting panel. It may have been useful to triangulate the documentary analysis with interviews with HIT applicants in the same way that interviews were conducted with the designers of the model itself. However, time and resource constraints militated against such an approach. Furthermore, given the interest in the HITs, many of the HIT directors and members have been accessed several times for research and evaluation purposes and may have found another research invitation burdensome. While this study is not an evaluation of the HITs’ performance, the data generated give some indication of where a formal evaluation of such structures may need to be targeted.

## Conclusions

By tracing the process- and outcomes-related themes underpinning the development of the HIT model, we have been able to demonstrate how a local broad-based alliance between NHS providers and commissioners, universities and local government, not part of an elite AHSC, has been able to design a flexible structural partnership to support a growing number of interprofessional, cross sectorial and cross-organisational teams operating in public health and service delivery for long-term conditions. ‘Whole system’ engagement, requiring the active recruitment of all those who have a stake in the area of practice being considered, and collaboration, understood as a range of internally and externally orientated strategies to enable coproduction were identified as the process themes. System-level integration and innovation were identified as outcomes with far reaching impacts on population health and service delivery. Detailed descriptions of emerging organisational models of structural partnerships - an example of which is provided in this article - and the identification of their underlying social and organisational processes are likely to be valuable to policy makers and senior leaders in the UK health and care economy, those involved in setting up similar partnerships and teams, and health services researchers in the social and implementation sciences with an interest in their evaluation. The HIT model is presented here to facilitate its translation to other localities.

## Abbreviations

AHSC, Academic Health Sciences Centre; BHP, Bristol Health Partners; CCG, Clinical Commission Group; CLAHRC, Collaborations for Leadership in Applied Health Research and Care; HIT, Health Integration Team; NHS, National Health Service
